# A Variant Carbapenem Inactivation Method (CIM) for *Acinetobacter baumannii* Group with Shortened Time-to-Result: rCIM-A

**DOI:** 10.3390/pathogens11040482

**Published:** 2022-04-18

**Authors:** Dieter Mitteregger, Julian Wessely, Ivan Barišić, Branka Bedenić, Dieter Kosak, Michael Kundi

**Affiliations:** 1 Laboratory Dr. Kosak, Dr. Reckendorfer and Partner, Department of Clinical Microbiology, 1090 Vienna, Austria; julian2012@hotmail.com; 2 Molecular Diagnostics, Center for Health & Bioressources, AIT Austrian Institute of Technology GmbH, 1210 Vienna, Austria; ivan.barisic@ait.ac.at; 3 School of Medicine, University of Zagreb, 10000 Zagreb, Croatia; bbedenic@mef.hr; 4 Clinical Department of Clinical and Molecular Microbiology, University Hospital Center Zagreb, 10000 Zagreb, Croatia; 5 Laboratory Dr. Kosak, Dr. Reckendorfer and Partner, Department of Clinical Pathology, 1090 Vienna, Austria; dr.kosak@dr-kosak.at; 6 Department of Environmental Health, Medical University of Vienna, Center for Public Health, 1090 Vienna, Austria; michael.kundi@meduniwien.ac.at

**Keywords:** carbapenem-resistant *Acinetobacter baumannii*, novel antibacterial agents, antimicrobial resistance, molecular mechanisms, phenotypic carbapenemase detection, rapid diagnostic test, nosocomial infection, infection prevention and control, OXA-type carbapenemases, carbapenem inactivation method

## Abstract

Carbapenem-resistant *Acinetobacter baumannii* group organisms (CRAB) are challenging because the choice between targeted, new antibiotic drug options and hygiene measures should be guided by a timely identification of resistance mechanisms. In CRAB, acquired class-D carbapenemases (CHDLs) are active against meropenem and imipenem. If PCR methods are not the first choice, phenotypic methods have to be implemented. While promising, the carbapenemase inactivation method (CIM) using meropenem-hydrolysis is, however, hampered by poor performance or overly long time-to-result. We developed a rapid CIM (rCIM-A) with good performance using ertapenem, imipenem, and meropenem disks, 2-h permeabilization and incubation with the test strain in trypticase soy broth, and a read-out of residual carbapenem activity after 6 h, and optionally after 16–18 h. Using clinical isolates and type-strains of *Acinetobacter* (*n* = 67) not harboring carbapenemases (*n* = 28) or harboring acquired carbapenemases (*n* = 39), the sensitivity of detection was 97.4% with the imipenem disk after 6 h at a specificity of 92.9%. If the inhibition zone around the ertapenem disk at 6 h was 6 or ≤26 mm at 16–18 h, or ≤25.5 mm for meropenem, the specificity was 100%. Because of the high negative predictive value, the rCIM-A seems particularly appropriate in areas of lower CRAB-frequency.

## 1. Introduction

The genus *Acinetobacter* represents Gram-negative rods or coccobacilli with a tendency to resist destaining, non-motility in liquid media (while surface-bound motility may be observed), catalase-positive, oxidase-negative, carbohydrate non-fermentative, chemo-organotrophic, and aerobic metabolism [1]. 

*A. calcoaceticus*, *A. baumannii*, *Acinetobacter* genomic species 3 (later *A. pittii* [2]), and *Acinetobacter* genomic species 13 *sensu* Tjernberg and Ursing “TU”, 1989 (later *A. nosocomialis* [3]), are very closely related and difficult to distinguish from each other by phenotypic properties, while biochemical differentiation from other members of the genus is possible [4]. It has been proposed by Gerner–Smidt et al. to refer to these species as the *A. calcoaceticus-A. baumannii* complex (ACB complex) [5,6]. Genetic studies support that the ACB complex represents a phylogenetically well-defined subgroup (clade) within the genus (cited in [7]). Since the recognition of the ACB complex, many new *Acinetobacter* species have been described, among which the clinically relevant and phylogenetically closely related *Acinetobacter seifertii* [7] and *Acinetobacter lactucae* [8] (heterotypic syn. *Acinetobacter dijkshoorniae* [9]) have been included in the complex. 

As *A. calcoaceticus*—in contrast to the other members of the taxonomically valid ACB complex—very rarely, if ever, causes clinically relevant infections, the clinically oriented *Acinetobacter baumannii* group (ACB group) has been coined, excluding *A. calcoaceticus* [10,11,12].

The clinical role of the members of the ACB group resemble each other; however, *A. baumannii* is more often associated with multidrug resistance and endemic spread in hospitals and with higher mortality among patients with systemic infections in case of carbapenem resistance [13,14]. In the United States in 2017, 8500 estimated cases and 700 estimated deaths occurred due to carbapenem-resistant *A. baumannii* (CRAB) [15]. Rice [16] reported *A. baumannii* in line with *Enterococcus faecium*, *Staphylococcus aureus*, *Klebsiella pneumoniae*, and *Pseudomonas aeruginosa* as the “ESKAPE” pathogens to emphasize that they effectively “escape” the effects of antibacterial drugs. The infection frequency is similar across Europe and Central America [17,18,19,20,21,22], while being even higher in Southeast Asia, India, and some countries in South America [23,24,25,26,27,28]. Accordingly, the WHO published a list of bacteria for which new antibiotics are urgently needed, and listed CRAB as critical and of highest priority [29]. 

The general resistance of *Acinetobacter* spp. to antibiotics stems in part from the very small number and permeability of porins in its outer membrane and in addition from multidrug efflux pumps [30]. Moreover, these bacteria have the capacity to rapidly acquire additional genetic entities for resistance even from other bacterial species [31,32].

*Acinetobacter* spp. isolates have shown a complex interaction of multiple mechanisms of resistance to carbapenems, with the production of naturally occurring oxacillinases (carbapenem-hydrolyzing class D ß-lactamases, CHDLs), grouped into closely related enzymes [33]. While *A. baumannii* harbors OXA-51 (OXA-51-Like Group), a chromosome-based enzyme intrinsic to the species, other CHDLs are acquired. The predominant CHDLs (OXA-23-Like Group, OXA-24 (syn. OXA-40)-Like Group, OXA-51-Like Group, OXA-58- Like Group, OXA-143-Like Group, and OXA-235-Like Group) are responsible for the majority of phenotypic resistance to carbapenems [12,33,34,35,36,37]. These Ambler-class D ß-lactamases are not very active carbapenemases, and some hydrolyze imipenem and ertapenem more actively than meropenem [38,39,40,41,42]. The presence of an insertion sequence (IS) element, such as IS*AbaI* and IS*Aba9*, increases the expression of the carbapenemase significantly, resulting in clinical carbapenem resistance [43,44,45]. Although the CHDLs listed above are described mainly in *A. baumannii*, they do spread to other *Acinetobacter* species [35,46,47]. *Acinetobacter* sp. can also acquire Ambler classes A and B carbapenemases (recently summarized in [48]).

Based upon the prevalence, morbidity and mortality impact of CRAB infections, several organizations, e.g., the German Commission for Hospital Hygiene and Infectious Disease Prevention (KRINKO) at the Robert Koch–Institute (RKI) [49,50,51] and the WHO [52], have established guidelines for the prevention, containment, and detection of nosocomial spread. Testing for carbapenemases should be considered as a routine in all microbiology laboratories as well as the reporting to health authorities, which is explicitly recommended [49,53].

With the availability of new antibiotics effective against bacteria producing carbapenemases of distinct Ambler classes, it is of particular importance to rapidly inform the clinicians about the presence and Ambler class of the detected carbapenemase [48,54,55,56,57]. In contrast to *Enterobacterales* or *P. aeruginosa*, carbapenemase-producing *Acinetobacer* spp. almost always harbor specific CHDLs. Simply knowing about carbapenemase production may therefore be sufficient. In turn, a positive result of genetic analysis without phenotypic testing may be problematic, especially in *A. baumannii,* as intrinsic OXA-51 ß-lactamases without insertion sequence-associated overexpression and mutations altering the substrate-specificity have only minimal effect on carbapenem susceptibility [3,12,58]. 

Criteria for laboratory selection of a carbapenemase detection test on isolates or clinical samples encompass epidemiology, diagnostic performance, labor intensity, complexity, and cost. Short turnaround times are important in patient management [55]. Assays divide into phenotypic and genotypic ones. 

Nucleic acid-based assays are sensitive, specific, and may have same-day turnaround time, but (except for the remaining demand of whole genome sequencing) would not detect the emergence of new or previously uncommon carbapenemase genes and are typically associated with high costs [59,60]. 

Phenotypic tests reveal carbapenemase activity either by the uninhibited growth of a carbapenem-sensitive reporter strain exposed to a potentially degraded carbapenem, by visualized carbapenem hydrolysis, or by mass changes detected by matrix assisted-time of flight (MALDI-TOF) mass spectrometry (MS). There are currently no specific inhibitors of the prevalent *Acinetobacter*-associated CHDLs. 

The 2017 European Committee on Antimicrobial Susceptibility Testing (EUCAST) guideline for the detection of resistance mechanisms, however, states that none of the existing phenotypic methods yield satisfactory results in the ACB group [53]. For more advanced methods such as MALDI-TOF MS, widespread implementation seems unfeasible due to complex processing and interpretation difficulties [61]. In *Enterobacterales,* the carbapenem inactivation method (CIM) [62,63] has been considered favorable in a modified version (mCIM) [64] with respect to sensitivity (97%) and specificity (99%), and was added to the Clinical and Laboratory Standards Institute M100 document [65]. In contrast, in the corresponding EUCAST document the CIM is mentioned as a possible alternative in *Enterobacterales*; however, with the accompanying information on the uncertain negative predictive value and the “disadvantage” of at least 18 h of time-to-result [53]. 

Because of the lack of accurate and rapid phenotypic methods for carbapenemase detection in *Acinetobacter*, variations of the classical CIM method [62] have been tested [64,66,67,68,69,70,71]. Performance characteristics of which are summarized in Table 1. 

The comparability of results is complicated by different definitions applied by various studies of what exactly is intended to be revealed by the CIM. These definitions span a spectrum from the demonstration of the activity of acquired carbapenemases (van der Zwaluw et al., 2015, Uechi et al., 2017, Simner et al., 2018, Jing et al., 2018, Vu et al., 2020, Howard et al., 2020, Cui et al., 2020) to the demonstration of the activity of chromosomal OXA-51-family, with (Liu et al., 2018, Uechi et al., 2019) or without associated insertion sequence (in Yamada et al., 2020). Since the objective of CIM was to reveal information relevant to infection control, choice of therapy, and epidemiology, supplementing the results of standard susceptibility testing, the authors of the present study retain the original intention [62,66] to demonstrate the presence of acquired carbapenemases and provide the accordingly transformed results of other studies in Table 1 (bold letters), where appropriate. 

**Figure 1:**
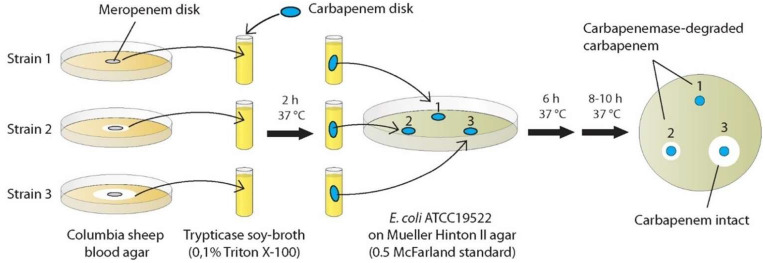
CIM using three carbapenem disks (ertapenem, imipenem, meropenem) incubated in a test strain suspension of 0.1% (vol/vol) Triton^TM^ X-100 in trypticase soy-broth and shortened time-to-result: rCIM-A. Starting point: agar disk-diffusion test of antimicrobial susceptibility-testing (meropenem disk). In routine-application six pre-incubated carbapenems disks (for two isolates) can be tested simultaneously on one read-out agar plate.

Due to the promising but still not standardized CIM methods applied so far, we conducted experiments applying another modified CIM method with the question of whether this procedure results in an early (~8 h) detection of carbapenemase production with high sensitivity and high reproducibility. This novel method consisted of advantageous features of existing protocols, which we combined and complemented with previously untested approaches: rCIM-A (Figure 1). 

## 2. Results

### 2.1. Exclusion of Strains Having Lost Genetic Resistance Determinants

First, a phenotypic evaluation of the strains was conducted by disk diffusion testing of meropenem and imipenem following EUCAST guidelines [54]. Based on these results, three strains (ACB6R, ACB8R, ACB9R) differed from their molecular genotypes with their susceptibility results and thus were excluded because of the probable loss of genetic resistance determinants. With respect to the possible enhancement of carbapenemase production, other research groups performed subculture in the presence of a meropenem disk in such cases; this was not feasible in the present study, since this procedure had been already an element of the study protocol.

### 2.2. Revelation of a New Category of Positive CIM Results: “Carryover Microsatellites”

**Figure 2:**
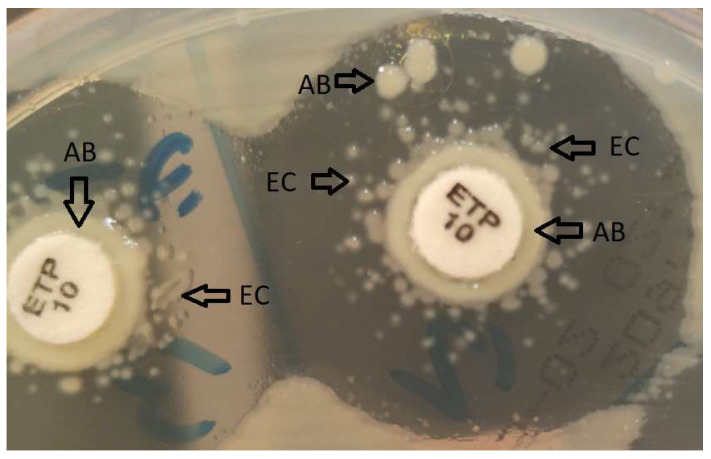
“Carryover microsatellites” of the indicator strain (*Escherichia coli* ATCC 25922, EC) surrounding a carryover growth (*Acinetobacter* spp. test strain, AB); ertapenem 10-µg potency disk, ETP 10.

We observed a, perhaps interesting, currently not yet reported phenomenon, which turned out to be of importance in the detection of positive CIM results in some cases. Around carbapenem disks previously incubated in suspensions of *A. baumannii* group test strains harboring acquired carbapenemase genes, characteristic carryover growth of the test strain sometimes occurred, despite careful wiping off excess suspension when removing the disk. In test strains harboring acquired carbapenemases surrounding the carryover growth were scattered colonies of the indicator strain which resembled the “microsatellite phenomenon” of *Haemophilus* spp. in the vicinity of colonies of hemolysin-producing *Staphylococcus* sp. on 5% sheep blood agar. The taxonomical identity of the colonies was revealed by MALDI-TOF MS. In these cases, the colonies of the indicator strain did not appear adjacent to the disk or originate from the periphery of the inhibition zone but were “satelliting” the carryover growth (Figure 2). Importantly, this phenomenon was interpreted as an effect of hydrolysis of residual carbapenem radiating from the pre-incubated test-disk, brought about by the carryover growth of a carbapenemase-positive test strain. Hence, even in the case of inhibition zone diameters above our calculated study cut-offs for positive results, these test strains could be recognized as true positives anyway. 

### 2.3. Performance of Phenotypic Detection of Acquired Carbapenemases in Acinetobacter spp. by the rCIM-A Protocol

#### 2.3.1. Intra-Observer Variability in the Single-Carbapenem Approaches 

As revealed by substance-/incubation time-specific coefficients of variation, every single approach was characterized by a very good reproducibility in triplicates, as <15% variation is considered a limit and <5% characterizes a very good test reproducibility (Table 2 and Table 3). Moreover, certain approaches performed significantly different from others, as indicated by the *p*-values (*p* significant at <0.05). Since CIM procedures in strains with or without acquired carbapenemases represent distinct categories, the analysis of reproducibility was conducted separately. Indeed, as may be expected, there was slightly more variation in the category of strains harboring no acquired carbapenemases. In addition, different approaches performed pronouncedly different with strains harboring acquired carbapenemases, while there was no such observation in the other category. In accordance with earlier reports of the weak performance of meropenem in CIM for *Acinetobacter* spp. [40,62,66], our results showed an at least 10-fold variation in comparison with imipenem in *Acinetobacter* sp. 

#### 2.3.2. Cut-Offs for Positive Results and Assay Parameters Adjusted to Maximum Test Performance vs. Emphasizing Sensitivity

Aside from applications of carbapenemase detection methods in the areas of epidemiology and public health, timely notification of the presence of carbapenemases in order to inform selection of appropriate last generation drugs and to institute adapted hygiene measures is critical for patient as well as overall health. We therefore provided alternative evaluations of single-carbapenem CIM-test performances for either more emphasis on the sensitivity than the specificity (weighting false negatives to false positives 2:1) or cut-offs targeting maximum test performance with equal weight to the sensitivity and specificity (represented by the Youden index). Table 4 shows the alternative approach-specific cut-offs and associated sensitivities and specificities. By nature, in emphasizing sensitivity, assay specificity falls, particularly in the case of ertapenem (specificity: 46.4%). Conversely, such performance is characteristically associated with particular high specificity if cut-offs are chosen according to maximization of the Youden index (specificity for ertapenem: 100%). Meropenem behaved similarly, though less pronounced, while equally high sensitivity and specificity were reached by the use of imipenem. 

Using rCIM-A correct results were obtained in up to 96% of cases as early as 6 h from the start of the disk-incubation with the read-out strain. Imipenem revealed best performance with respect to accuracy and rapidity. 

**Figure 3:**
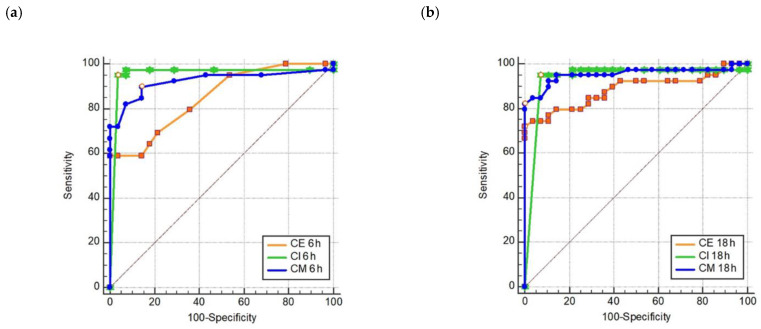
Receiver operating characteristic (ROC) showing single-CIM test performance in 6 h (**a**) and 16–18 h (**b**); rCIM-A with ertapenem (CE)/imipenem (CI)/meropenem (CM) disk read-out after 6 and 16–18 h.

All single carbapenem-CIM tests were highly discriminative for the presence of acquired carbapenemases as revealed by the individual receiver operating characteristic (ROC) statistics (Figure 3). Area under the curves (AUC) were (except for ertapenem) never below 0.92 at significance levels of *p* < 0.0001.

As test performance is always relative to the prevalence of the event in focus, we calculated positive/negative predictive values (PPV/NPV) of single CIM-tests according to the global spectrum of CRAB distribution. For all single approaches, the NPV was very good to acceptable for a prevalence up to 70% (Table 5). Conversely, the PPV was very good to acceptable (except for ertapenem) down to a prevalence of 30% (Table 6).

## 3. Discussion

Given the advent of last generation anti-infective drugs, which require knowledge of resistance mechanisms of the causative organisms [48,54,57] as well as the development of effective worldwide strategies against nosocomial transmission of resistant pathogens [52], the need for rapid and accurate laboratory methods for the identification of such isolates, even in low-resource settings, has been recognized as a priority in the last years. 

Existing protocols never resulted in simple methods of good performance with time-to-result shorter than 16–18 h (cf. Table 1).

In the present study, we developed the first 8 h/“within-a-day” CIM method for routine-diagnostics application, called rCIM-A, enabling correct results within this shortened turn-around time in up to 96% of cases. Ertapenem, meropenem, and imipenem disks can be used simultaneously, as all reagents needed are together below 1 Euro per test and the processing is very simple with minimal hands-on time. It could be expected that the assay performance would not be strongly negatively affected if the three potency disks were incubated in one and the same strain suspension. The evaluation of all disks after 6 h of incubation resulted in a sensitivity near 100%. If there was no inhibition zone around the ertapenem disk after 6 h, the specificity of the positive result was 100%. This represented the main value of ertapenem disk inclusion. The highest sensitivity at 6 h was provided by the imipenem disk (97.4%) and continuing the incubation of any disk added nothing to sensitivity. If there was an inhibition zone around the ertapenem disk at 6 h, checking the ertapenem or meropenem inhibition zones (≤26 or ≤25.5 mm for positive results, respectively) at 16–18 h confirmed a negative result at 100% specificity.

In the following, we compiled an interpretative overview of CIM history and variations and compared the design of the rCIM-A, subsequently enabling the performance characteristics summarized above.

Van der Zwaluw et al. [62], in their seminal work, used a methodological principle introduced by Masuda et al. in the year 1976 [76] to demonstrate the existence of diffusable ß-lactamases of Gram-negative bacteria. Since this screening method for the detection of ß-lactamase activity represented a laborious diagnostic procedure, it was later simplified by the so-called Hodge test [77]. Thereby, the principle was maintained to reveal the production of a hydrolyzing enzyme, released by a test strain into the growth agar, by the growth of a sensitive indicator strain towards a centered antibiotics disk. As the Hodge test suffered from the subjective interpretation of results and performance issues, it was in recent times no longer recommended [53,78]. In contrast, the novel CIM by van der Zwaluw et al. moved the hydrolysis step from growth agar to a preceding incubation step of a carbapenem disk in a suspension of the test strain. In the case of a carbapenemase-producing test strain, the potency loss of the disk was again read-out by the growth of a sensitive indicator strain [62]. The characteristic features of this original method were suspending a 10 µL inoculation loop of culture in 400 µL of water and incubating a 10 µg meropenem disk in preparation for later biological analysis of the remaining potency. The authors concluded, with respect to *A. baumannii*, which difficulties detecting low-level carbapenemase activity represented a limitation of the CIM. 

In order to overcome the low sensitivity of CIM in *Acinetobacter* spp. due to the low-level carbapenemase activity and low membrane permeability [66] Uechi et al., 2017 used Tris-HCl buffer instead of trypticase soy broth (TSB) to extract carbapenemases. At a typically long time-to-result and acceptable sensitivity, the specificity let room for improvement [72]. Tris-HCl was later shown by others to degrade meropenem rather quickly [69].

According to the modified (m)CIM, working fine for *Enterobacterales* [64], which was added later as a CLSI method in this application [65], Simner et al. incubated a 10 µg meropenem disk in TSB, but found—instead of a 1 µL— only a 10 µL inoculation loop of culture bringing about a modest increase in sensitivity in *A. baumannii* strains. Due to the, therefore, lower specificity in this approach, the performance was described as less than ideal for *A. baumannii* [66]. 

Liu et al. increased sensitivity by the permeabilization of *A. baumannii* complex with 0.1 % (vol/vol) Triton^TM^ X-100 in TSB and using a 10 µL inoculation loop of culture. The maximized performance demonstrated in this study [79] could not be reproduced by Yamada et al. [80] using various CHDL-producing strains. The assessed performance depends on the definition of carbapenemase-positive strains as shown in Table 1. However, investigating the CRAB consisting of 93% OXA-23 strains and using a modification of Liu’s TCIM by incubating a 10 µg meropenem disk for 2 h in 400 µL TSB containing 0.1 % (vol/vol) Triton^TM^ X-100 and reading results after 16–18 h, Fan et al. recently reported sensitivity and specificity of 100%, respectively [71]. 

Jing et al. deviated substantially from what was introduced as CIM by van der Zwaluw et al., since they, firstly, exchanged the disk-incubation step in a suspension of the test strain by directly smearing 1–3 colonies of the test strain onto the carbapenem-disk and, secondly, shifted the incubation phase of test strain and disk to the 16–18-h agar diffusion phase with the indicator strain (inoculated at a 1:10 dilution of 0.5 McFarland standard suspension). As they did not report on test repetitions, it is currently unknown if the direct smear method of colonies reaches the high robustness and reproducibility of the original CIM [62]. In contrast to other approaches, this group used a 10-µg imipenem disk. Again, the reported maximized performance [73] could not be reproduced by Yamada et al. [69], and the reduced sensitivity in the hands of this group may have been partly due to the limited number of colonies used for the direct smear, of which some could have lost genetic resistance determinants. Further, for the original validation of sCIM only OXA-23 strains and one VIM-2 strain have been used [66], while Yamada et al. tested various CHDL producers [69]. 

Uechi et al. (2019) then tried to optimize their previous CIMTris-protocol further and were successful to some extent by using a 5 µg meropenem disk incubated for 1 h in a suspension of a 5 µL loopful (described as on-third of a 10 µL loop) of culture. Again, the time-to-result remained unabbreviated, and the authors did not address the question, if the delicate skill of measuring “one-third of a 10 µL loop” may translate into a method of high intra- and interobserver reproducibility [74]. The reversion of the evaluation criteria to the one used by this group in their previous CIM-publication demonstrates that the sensitivity gain was on cost of specificity (Table 1). 

Vu et al., interestingly, showed a dramatical increase in performance of mCIM [66] in *A. baumannii* (83% of acquired carbapenemase-genes were comprised of OXA-23 in this study) by reverting incubation time and volume (400 µL) back to the conditions introduced by van der Zwaluw et al. while revealing that keeping TSB as incubation medium is optimal over Tris-HCl and using an unusual big inoculum of two 10 µL loopfuls of bacteria. Even the long time-to-result of the CIM and mCIM protocols was kept [68]. 

Howard et al. reported the same approach differing in a refinement of sensitivity by using the prolonged suspension-incubation time of the mCIM (4 h) and of specificity by reducing the suspension-inoculum to the half of a 10 µL incubation loop. The long time-to-result of 18–24 h remained again unchanged, and we are unaware of the reproducibility of the method, as well as of the number of carbapenemase-negative *A. baumannii* strains included in the study [75]. 

Cui et al. presented a comparatively costly approach of seemingly shortened time-to-result by a CIM-related methodology, which reminds us—with respect to the laborious in-house concept—of Masuda’s 1976 screening method [80]. Their starting point is the overnight liquid culture of the test strain, i.e., not from the common agar diffusion susceptibility test. The rest-potency of a 10 µg imipenem disk is indicated by growth of *Bacillus stearothermophilus* 7453 on an in-house chromogenic plate at 60 °C. All CHDLs in this study were comprised of OXA-23 [70].

Yamada et al. showed that 0.5 M Tris-HCl as an incubation matrix rapidly degrades meropenem in the antibiotic disk, while no substrate was degraded using 3-morpholinopropane-1-sulfonic acid (MOPS), which was therefore chosen for LCIM. In this method, the concentration of Triton^TM^ X-100 was increased to lytic levels of 2% (vol/vol) in a 4-h incubation step with the disk, followed by a standard incubation time with the indicator strain. In an attempt to maximize sensitivity, this group reported on the detection of OXA-51-positive strains without IS*Aba1* [69], which clearly misses the point as those detections essentially just reveal the presence of *A. baumannii* per se and should thus be regarded as false-positives. 

Characteristic similarities and differences between existing and the novel method are briefly summarized below.

To make use of potential induction of the weak *A. baumannii* group carbapenemases colonies were taken from the edge of the inhibition zones [66] around meropenem disks used for routine-AST. 

As smaller volumes were shown to reduce sensitivity at better specificity in *Acinetobacter* too much [66,67,75], the volume contained in a 10 µL inoculation loop was used for preparing a suspension in 400 µL TSB containing 0.1% (vol/vol) Triton^TM^ X-100. The mildly membrane-permeabilizing 0.1% (vol/vol) Triton^TM^ X-100 [67,71] plus TSB (carbapenem-protective, [68]) approach was chosen, since the disk incubation step in water-suspension [62] or TSB [66] did not result in sufficient bacterial-borne lytic activity [62] and Tris-HCl extraction was shown to degrade the carbapenem unspecifically [69]. 

Representing a new CIM modification, a 10 µg potency disk of ertapenem, meropenem, and imipenem was added to test-strain suspensions. One may ask why one should deviate from the original CIM protocol [62,66,67] by using imipenem or, in particular, the clinically ineffective ertapenem in *A. baumannii*-CIM. Importantly, the most prevalent carbapenemases in *A. baumannii* (CHDLs) are low-activity enzymes, being comparatively difficult to detect phenotypically; to this end these enzymes should therefore be combined with substrates, they do most actively convert. Conversely, the in vitro or clinical effectivity of the carbapenemase-substrates (assay-carbapenems) on *A. baumannii* have no impact on the test performance at all. The carbapenem does not have to inwardly cross the *Acinetobacter* outer membrane, but awaits, on the contrary, the exit of carbapenemases. The read-out strain (*Escherichia coli* ATCC 25922) only has to be susceptible. Imipenem [72] and, more, ertapenem [42] are most effectively hydrolyzed by CHDLs and were, for sensitivity and assay-rapidity reasons, integrated in the rCIM-A protocol. 

Due to above-described optimizations a read-out was performed as early as after 6 h and after additional 8–10 h of incubation, enabling the potential shortest CIM protocol suitable for routine-use so far (Figure 1). 

Although the substrate-specific activities of the predominating Ambler class D carbapenemases in *Acinetobacter* spp. have been known for a long time, the present study describes, irrespective of two quite departing variations [70,73], the only classical, and hence robust and straightforward, CIM method using imipenem. The inclusion of two other carbapenem potency disks in the protocol may be advantageous in case of carbapenemases of the Ambler classes A and B, which also occur in the *A. baumannii* group with varying prevalence globally. To our knowledge, ertapenem has never been tested in a CIM approach previously. 

Because of the high NPV revealed, the rCIM-A seems particularly appropriate in areas with lower CRAB frequencies and when PCR tests (NAATs) are not available because of high costs, required technical skills, and/or laboratory compartments. In addition, PCRs are not free of hypotheses and may not detect new or locally rare genetic variants. 

The newly described phenomenon of “carryover microsatellites” may be helpful even in other variants of CIM in order not to overlook true-positive results.

Further studies may investigate the ability of included specific inhibitors of Ambler class A and B carbapenemases to discriminate these from CHDLs, in order to inform appropriate therapeutic decisions in areas where the class spectrum of carbapenemases in *A. baumanii* group is more diverse.

As for every diagnostic laboratory method, the validation in a clinical study addressing the clinical benefit would be desirable. 

## 4. Materials and Methods

### 4.1. Strains of Acinetobacter spp.

Strains of *A. baumannii* group not harboring acquired carbapenemases (*n* = 28) were isolated from clinical specimens submitted to the Laboratory Dr. Kosak, Dr. Reckendorfer and Partners in the year 2021 by physicians and private hospitals in eastern Austria, from clinical specimens were sent to the University Hospital Center Zagreb in Croatia, and from the German Collection of Microorganisms and Cell Cultures (DSMZ), respectively. Strains of *A. baumannii* harboring acquired carbapenemases (*n* = 39) were collected and genetically analyzed at the University Hospital Center Zagreb in Croatia. Type strains from the UK (National Collection of Type Cultures) or German (DSMZ) collections were also used, according to the results of genetic testing. After cryopreservation and thawing before use in the study protocol, all isolates and strains (*n* = 67) were subcultured and checked for purity and identity by MALDI-TOF MS (Bruker Daltonics, Bremen, Germany) and genetic testing (where performed). 

This study does not include any patient information; hence, approval from Institutional Review Board was not necessary.

### 4.2. Assigning Strains to the Sample Not Harboring/Harboring Acquired Carbapenemases 

Clinical isolates were tested according to EUCAST guidelines [54]. Isolates fully susceptible to meropenem and imipenem were considered to have no acquired carbapenemases [51,56] (Table 7). Otherwise, genetic analysis was performed by multiplex PCR or whole-genome sequencing. Relevant genetic resistance determinants are listed in Table 7. 

#### 4.2.1. Molecular Detection of Resistance Genes

The detection of carbapenem-resistant genes was determined by PCR. To amplify the genes conferring resistance to β-lactams including carbapenemases of Ambler class A (*bla*_KPC_), Ambler class B (metallo-β-lactamases, MBLs: *bla*_VIM_, *bla*_IMP_, and *bla*_NDM_), and Ambler class D (carbapenem hydrolyzing oxacillinases, CHDLs: *bla*_OXA-48_), we used primers and cycling conditions, previously described. Genes encoding KPC, MBLs (*bla*_VIM_, *bla*_IMP_, *bla*_SIM,_ and *bla*_NDM_) and CHDLs (*bla*_OXA-51-like_, *bla*_OXA-23-like_, *bla*_OXA-24/40-like_, *bla*_OXA-58-like_, and *bla*_OXA-143-like_) were amplified by PCR using protocols and conditions as described previously [81,82]. 

#### 4.2.2. Whole-Genome Sequencing

A subset of the clinical isolates was analyzed using whole-genome sequencing (WGS). The bacterial genomes were sequenced using the IonTorrent PGM platform (Life Technologies, Carlsbad, USA) in accordance with the manufacturer’s instructions. The Ion Xpress Plus Fragment Library Kit was used to enzymatically shear 100 ng of the genomic DNA. The target fragment size was 400 bp. Subsequently, the fragmented DNA was processed using the Ion DNA Barcoding kit (Life Technologies), and its size was selected using the E-Gel SizeSelect 2% Agarose kit (Life Technologies). The size distribution of the DNA fragments was analyzed using the High Sensitivity kit (Agilent, Santa Clara, USA). Further sample processing was performed using the Ion OneTouch kit (Life Technologies). Finally, the amplified DNA was sequenced using the 318 chip (Life Technologies). The single reads obtained were de novo assembled using MIRA 3.9.9, which is a part of the Assembler plugin on the Ion Torrent server. The contigs were analyzed using the ResFinder version 4.1. web-service [80].

### 4.3. Optimization of Ertapenem Experimental Conditions

In a pilot trial, we investigated a specific, simplified protocol for CIM using only an ertapenem disk. We thus evaluated incubation times in the test strain suspensions from 30 min up to two hours and varied the matrix with either water or TSB, with or without addition of 0.1% (vol/vol) Triton^TM^ X-100 (see Supplementary Table S1). A two-hour incubation and addition of Triton^TM^ X-100; however, turned out to be critical in our hands (see Supplementary Table S1 and Supplementary Figure S1). The following steps and read-out were identical to the rCIM-A protocol. 

### 4.4. Phenotypic Detection of Acquired Carbapenemases by Application of a New Modification of the CIM Using Three Carbapenem Disks in a Rapid Format: Rapid CIM for Acinetobacter spp. (rCIM-A)

Strains were subcultured twice on BD^TM^ Columbia Agar with 5% Sheep Blood (Becton Dickinson GmbH, Heidelberg, Germany) in order to disperse genetic resistance determinants [66]. To induce the carbapenemase production, where possible, the second passage was conducted in the presence of a meropenem disk (10 µg, Mast Group Ltd., Liverpool, UK), since the success of this detection method relies on the hydrolysis of carbapenems by enzymes of predominantly week activity [82]. This approach is reasonable, too, as the starting point of carbapenemase testing in the diagnostic laboratory will mostly be the finding of carbapenem resistance in a disk diffusion test. 

Hence, colony material was taken from the edge of the inhibition zone around the meropenem disk for further processing [80] (Figure 1). As smaller volumes were shown to reduce sensitivity and specificity in *Acinetobacter* spp. [66,67,75], the volume contained in a sterile 10 µL inoculation loop was used for preparing a suspension in 400 µL BD^TM^ trypticase soy broth containing 0.1% Triton^TM^ X-100 (VWR-Chemicals, Radnor, Pennsylvania), a detergent, commonly used to permeabilize the membranes of living cells. TSB protects the carbapenem contained in the potency disks better from activity loss than other matrices during the consecutive incubation step [68], which is critical for test specificity. Permeabilization of the outer *Acinetobacter* spp. membrane, which has intrinsic low permeability [66], facilitates the release of carbapenemases from the periplasmatic space into the TSB during the incubation step with the carbapenem disk and thus fosters test sensitivity [79] as does the smaller incubation volume [62,68,75], which is 2.000 µL in the mCIM [66]. 

Representing a new CIM modification, a 10 µg potency disk of ertapenem, meropenem, and imipenem was added to a separate tube of test strain suspension for a 2-h incubation at 35 +/− 2 °C [66], which previously proved sufficient [68]. 

In preparation of the read-out step, a suspension of the CIM indicator organism *Escherichia coli* ATCC 25922, with a turbidity equivalent of a 0.5 McFarland standard was streaked onto a BD BBL Mueller–Hinton agar (MHA) plate according to the EUCAST disk diffusion method [54,80]. The disks were removed from the tubes at the end of the incubation time using a sterile 10 µL inoculation loop, which was dragged along the edge of the tube during removal to leave excess liquid, and were placed on the MHA plate [64]. One standard MHA plate can be used for the placement of up to 6 test-disks. Against the background of several potential efficiency optimizations read-out was performed after 6 h and after additional 8–10 h of incubation at 35 +/− 2 °C in ambient air, in total corresponding to 16–18 h of incubation.

All strains were tested in triplicates, each starting from the preparation of the bacterial suspension. A positive control (molecular detection of an acquired carbapenemase and a phenotypic carbapenem resistance) and a negative control (fully susceptible phenotype) were included in every test round and had to reveal expected results. 

Plates were read by a trained biomedical analyst using a caliper rule. Colonies within the inhibition zone were regarded as significant. When multiple colonies were observed growing within a zone of inhibition, the result was interpreted as positive. In the case of minimal growth of a few pinpoint colonies, the test was repeated, and the repeat result was used for data analysis [66]. A narrow ring of growth surrounding the test-disk was regarded as a carryover of the test organism from the suspension and was ignored [64].

### 4.5. Statistical Methods

Because the number of strains available to us was fixed, we performed no formal sample size calculation; however, we did a power analysis considering the area under the curve in the ROC analysis as the outcome of interest. We defined an AUC of 70% as a margin that should be detected at a significance level of 5%. The power to detect this effect in our study was 82%.

For each strain, the coefficient of variation (CV) was computed for the triplicates. Results were square root transformed and means and standard deviations of these values were computed separately for strains with and without acquired carbapenemase. The methods were compared with respect to the CV by Student’s *t*-test. ROC analysis was performed on a random sample of one value of the triplicates for each strain. The sensitivity and specificity were computed based on two criteria: first, at the minimum of the cost function computed for a weight ratio of false negatives to false positives of 2:1 (hence optimizing sensitivity), and second, at the maximum of the Youden index. Positive and negative predictive values were computed for the range of prevalence of carbapenemase positivity in the population of specimens for which the test is required of between 10 and 90% (accounting for the wide variation reported in the literature). Statistical analyses were performed using Stata 17.0 (StataSoft, College Station, TX, USA). Graphics of ROC curves were plotted by MedCalc 20.23 (MedCalc, Ostend, Belgium).

## Figures and Tables

**Table 1:** Synopsis of CIM variants for *Acinetobacter* spp.

First Authors, Year	CIM Brand	Sensitivity (%)/Specificity (%) According to Study Authors Acquired Carbapenemases		Incubation + Time-to-Result (h)	Ref.
van der Zwaluw, 2015	CIM	10/12 (83)/5/6 (83)	10/12 (83)/5/6 (83)	2 + 6 or over night	[62]
Uechi, 2017	CIMTris	27/29 (93.1)/13/15 (86.7)	27/29 (93.1)/13/15 (86.7)	2 + 18	[72]
Simner, 2018	mCIM	79.8 (mean)/52.9 (mean)	79.8 (mean)/52.9 (mean)	4 + 18–24	[66]
Liu, 2018	TCIM	83/83 (100)/69/69 (100)	**82/105 (78.1)/46/47 (97.9)**	4 + 18–24	[67]
Jing, 2018	sCIM	53/53 (100)/20/20 (100)	53/53 (100)/20/20 (100)	0 + 16–18	[73]
Uechi, 2019	CIMTrisII	34/35 (97.1)/20/22 (90.9)	**31/32 (96.9)/20/25 (80)**	1 + 18	[74]
Vu, 2020	Optimal mCIM	46/46 (100)/27/27 (100)	46/46 (100)/27/27 (100)	2 + 18–24	[68]
Howard, 2020 ^1^	iCIM	10/10 (100)/- ^2^ (100)	10/10 (100)/- ^2^ (100)	4 + 18–24	[75]
Cui, 2020 ^3^	CIM^B.S.^	18/19 (94.7) /5/5 (100)	18/19 (94.7) /5/5 (100)	0.5 + 3.5	[70]
Yamada, 2020 ^4^	LCIM	99/102 (97.1)/57/57 (100)	**62/64 (96.9)/58/95 (61.1)**	4 + 18–24	[69]

^1^ Revealed sensitivity (%)/specificity (%) for CIM, mCIM of 7/10 (70)/(100), 1/10 (10)/100; ^2^ number of carbapenemase negative strains not provided, but amount to 30, including strains of *P. aeruginosa;* ^3^ this method starts from overnight liquid culture, not from the common disk diffusion antimicrobial susceptibility test (AST); ^4^ revealed sensitivity (%)/specificity (%) according to the study authors for mCIM, sCIM, CIMTris, TCIM of 72/102 (70.6)/57/57 (100), 66/102 (64.7)/50/57 (87.7), 89/102 (87.3)/57/57 (100), 81/102 (79.4)/47/57 (82.5); according to acquired carbapenemases for mCIM, sCIM, CIMTris, TCIM of **57/64 (89.1)/76/95 (80), 55/64 (85.9)/74/95 (77.9), 61/64 (95.3)/66/95 (69.5), 61/64 (95.3)/65/95 (68.4)**; carbapenem inactivation method, CIM; Tris-HCl extraction CIM, CIMTris; modified CIM, mCIM; Triton CIM, TCIM; simplified CIM, sCIM; Tris-HCl extraction variant II CIM, CIMTrisII; in-house CIM, iCIM; CIM with *Bacillus stearothermophilus* 7453 chromogenic-agar read-out, CIM^B.S.^; lysate CIM, LCIM.

**Table 2:** Coefficient of variation (CV%) and *p*-values for the comparison of single-CIM-tests in strains with acquired carbapenemases by time-to-read-out; rCIM-A with ertapenem (CE)/imipenem (CI)/meropenem (CM) disk read-out after 6 and 16–18 h.

Method	CV%	CE 6 h	CE 18 h	CI 6 h	CI 18 h	CM 6 h
CE 6 h	0.4	-				
CE 18 h	0.3	0.696	-			
CI 6 h	0.03	0.021	-	-		
CI 18 h	0.02	-	0.033	0.891	-	
CM 6 h	1.9	0.084	-	0.003	-	-
CM 18 h	0.3	-	0.941	-	0.145	0.095

**Table 3:** Coefficient of variation (CV%) and p-values for the comparison of single-CIM-tests in strains without acquired carbapenemases by time-to-read-out; rCIM-A with ertapenem (CE)/imipenem (CI)/meropenem (CM) disk read-out after 6 and 16–18 h.

Method	CV%	CE 6 h	CE 18 h	CI 6 h	CI 18 h	CM 6 h
CE 6 h	2.0	-				
CE 18 h	1.9	0.930	-			
CI 6 h	3.6	0.100	-	-		
CI 18 h	1.7	-	0.377	0.028	-	
CM 6 h	2.0	0.904	-	0.101	-	-
CM 18 h	2.0	-	0.654	-	0.222	0.982

**Table 4:** Sensitivity and specificity (and 95% confidence intervals) of single CIM-tests, given 1:2 weighting of false-positive results to false-negative results and calculation at the minimum of costs and at the maximum of the Youden index by time-to-read-out; rCIM-A with ertapenem (CE)/imipenem (CE)/meropenem (CM) disk read-out after 6 and 16–18 h.

	At Cost Minimum			At Youden Index Maximum		
Method	Cut-Off	Sensitivity %	Specificity %	Cut-Off	Sensitivity %	Specificity %
CE 6 h	≤25	94.9 (82.7–99.4)	46.4 (27.5–66.1)	≤6	59.0 (42.1–74.4)	100.0 (87.7–100.0)
CE 18 h	≤28.5	92.3 (79.1–98.4)	46.4 (27.5–66.1)	≤26	71.8 (55.1–85.0)	100.0 (87.7–100.0)
CI 6 h	≤17	97.4 (86.5–99.9)	92.9 (76.5–99.1)	≤6	94.9 (82.7–99.4)	96.4 (81.7–99.9)
CI 18 h	≤21	94.9 (82.7–99.4)	89.3 (71.8–97.7)	≤6	94.9 (82.7–99.4)	92.9 (76.5–99.1)
CM 6 h	≤22	89.7 (75.8–97.1)	85.7 (67.3–96.0)	≤22	89.7 (75.8–97.1)	85.7 (67.3–96.0)
CM 18 h	≤26.5	94.9 (82.7–99.4)	85.7 (67.3–96.0)	≤25.5	82.1 (66.5–92.5)	100.0 (87.7–100.0)

**Table 5:** Negative predictive value of single CIM-tests (according to prevalence distribution); rCIM-A with ertapenem (CE)/imipenem (CI)/meropenem (CM) disk read-out after 6 and 16–18 h.

Prevalence	CE 6 h	CE 18 h	CI 6 h	CI 18 h	CM 6 h	CM 18 h
10%	98.8%	98.2%	99.7%	99.4%	98.7%	99.3%
20%	97.3%	96.0%	99.3%	98.6%	97.1%	98.5%
30%	95.5%	93.4%	98.8%	97.6%	95.1%	97.5%
40%	93.2%	90.0%	98.2%	96.3%	92.6%	96.2%
50%	90.1%	85.8%	97.3%	94.6%	89.3%	94.4%
60%	85.8%	80.1%	96.0%	92.1%	84.7%	91.8%
70%	79.6%	72.1%	93.9%	88.3%	78.1%	87.8%
80%	69.5%	60.1%	89.9%	81.5%	67.5%	80.8%
90%	50.3%	40.1%	79.9%	66.2%	48.0%	65.1%

**Table 6:** Positive predictive value of single CIM-tests (according to prevalence distribution); rCIM-A with ertapenem (CE)/imipenem (CI)/meropenem (CM) disk read-out after 6 and 16–18 h.

Prevalence	CE 6 h	CE 18 h	CI 6 h	CI 18 h	CM 6 h	CM 18 h
10%	16.4%	16.1%	60.4%	50.6%	41.1%	42.4%
20%	30.7%	30.1%	77.4%	69.7%	61.1%	62.4%
30%	43.1%	42.5%	85.5%	79.8%	72.9%	74.0%
40%	54.1%	53.4%	90.1%	86.0%	80.7%	81.6%
50%	63.9%	63.3%	93.2%	90.2%	86.3%	86.9%
60%	72.6%	72.1%	95.4%	93.3%	90.4%	90.9%
70%	80.5%	80.1%	97.0%	95.6%	93.6%	93.9%
80%	87.6%	87.3%	98.2%	97.4%	96.2%	96.4%
90%	94.1%	93.9%	99.2%	98.8%	98.3%	98.4%

**Table 7:** Carbapenem susceptibilities and ß-lactamases for strains with and without acquired carbapenemases.

Strain	Species	Inhibition Zone mm		EUCAST		Carbapenemases/ß-Lactamases
				S-I-R		
		MP	IP	MP	IP	
ACB1R	*A. baumannii*	6	6	R	R	OXA-24/40
ACB2R	*A. baumannii*	6	6	R	R	OXA-24/40
ACB3R	*A. baumannii*	6	6	R	R	OXA-24/40
ACB4R	*A. baumannii*	6	6	R	R	OXA-24/40
ACB5R	*A. baumannii*	6	6	R	R	OXA-24/40
ACB7R	*A. baumannii*	6	6	R	R	OXA-24/40
ACB10R	*A. baumannii*	6	6	R	R	OXA-24/40
ACB11R	*A. baumannii*	6	6	R	R	OXA-24/40
ACB12R	*A. baumannii*	19.9	23.1	I	I	OXA-24/40
ACB2	*A. baumannii*	6	6	R	R	OXA-24/40
ACB5	*A. baumannii*	6	6	R	R	OXA-23
ACB9	*A. baumannii*	6	6	R	R	OXA-58
ACB10	*A. baumannii*	6	6	R	R	OXA-58
ACB11	*A. baumannii*	6	6	R	R	OXA-58
ACB15	*A. baumannii*	6	6	R	R	OXA-58
ACB25	*A. baumannii*	6	6	R	R	OXA-24/40
ACB38	*A. baumannii*	6	6	R	R	OXA-58
ACB42	*A. baumannii*	6	17.7	R	R	OXA-58, OXA-107 ^1^, ADC-25 ^2^
AB41M	*A. baumannii*	7	11.6	R	R	OXA-23, OXA-66 ^1^, ADC-25 ^2^
AB43M	*A. baumannii*	6	6	R	R	OXA-72, OXA-66 ^1^, ADC-25 ^2^
AB44	*A. baumannii*	6	6	R	R	OXA-72, OXA-66 ^1^, ADC-25 ^2^
ACB46	*A. baumannii*	6	6	R	R	OXA-24/40
ACB47	*A. baumannii*	6	6	R	R	OXA-58
ACB70	*A. baumannii*	6	6	R	R	OXA-58
ACB72	*A. baumannii*	6	6	R	R	OXA-23
1475/18	*A. baumannii*	6	6	R	R	OXA-23, OXA-66 ^1^, ADC-25 ^2^
NCTC13305	*A. baumannii*	14.1	13.3	R	R	OXA-58, OXA-100 ^1^, ADC-25 ^2^
WS	*A. baumannii*	6	6	R	R	OXA-24/40
ACB39	*A. baumannii*	11.1	11.2	R	R	OXA-58
ACB19	*A. baumannii*	9.8	18.0	R	R	OXA-23
GIM	*A. baumannii*	6	6	R	R	GIM-1 ^3^, OXA-72, OXA-50 ^4^, OXA-69 ^1^, ADC-25 ^2^
DSM25645	*A. baumannii*	6	6	R	R	OXA-24/40
1966/18	*A. baumannii*	6	6	R	R	OXA-23, OXA-66 ^1^, ADC-25 ^2^
1306/18	*A. baumannii*	6	6	R	R	OXA-23, OXA-66 ^1^, ADC-25 ^2^
AB44921	*A. baumannii*	6	7.3	R	R	OXA-23, OXA-66 ^1^, ADC-25 ^2^
M/10639	*A. baumannii*	6	6	R	R	OXA-23, OXA-66 ^1^, PER ^5^, ADC-25 ^2^
5813/18	*A. baumannii*	6	9.8	R	R	OXA-23
4802colR	*A. baumannii*	6	6	R	R	OXA-23
1	*A. baumannii*	6	6	R	R	OXA-24/40, OXA-72, OXA-69 ^1^
46048	*A. lactucae*	28.5	31.3	S	S	-
47209	*A. baumannii*	22.8	29.9	S	S	-
1705	*A. pittii*	28.2	28.0	S	S	-
1997	*A. pittii*	26.1	31.7	S	S	-
47620	*A. pittii*	27.0	31.2	S	S	-
47591	*A. pittii*	26.0	33.3	S	S	-
46851	*A. lactucae*	27.6	28.5	S	S	-
ACB1	*A. baumannii*	13.5	19.4	R	R	OXA-51 ^1^
ACB12	*A. baumannii*	18.2	22.2	I	I	OXA-51 ^1^
48138	*A. pittii*	25.5	27.2	S	S	-
48911	*A. baumannii*	25.2	29.8	S	S	-
49593	*A. baumannii*	26.0	28.9	S	S	-
50018	*A. pittii*	26.4	29.0	S	S	-
51091	*A. pittii*	26.9	31.4	S	S	-
AB45M	*A. baumannii*	20.3	25.0	I	S	OXA-66 ^1^, ADC-25 ^2^
51237	*A. baumannii*	26.5	30.0	S	S	-
52037	*A. pittii*	25.7	27.6	S	S	-
52382	*A. pittii*	26.2	29.5	S	S	-
52977	*A. pittii*	25.1	26.7	S	S	-
53344	*A. baumannii*	25.5	31.3	S	S	-
54054	*A. pittii*	26.4	27.8	S	S	-
54135	*A. baumannii*	24.1	28.6	S	S	-
DSM102856	*A. nosocomialis*	26.9	31.1	S	S	ADC-25 ^2^
DSM30007	*A. baumannii*	23.6	31.4	S	S	OXA-98 ^1^, OXA-51 ^1^, ADC-25 ^2^
8314	*A. pittii*	24.0	29.1	S	S	-
55494	*A. pittii*	26.7	29.3	S	S	-
55525	*A. baumannii*	27.1	30.5	S	S	-
55705	*A. pittii*	24.4	28.2	S	S	-

^1^ OXA-51-Like Group; ^2^ ADC-type chromosomal Ambler class C (AmpC) extended-spectrum cephalosporinase; ^3^ metallo-ß-Lactamase (Ambler class B) from *P. aeruginosa*; ^4^ low-level chromosomal carbapenemase (Ambler class D) in *P. aeruginosa*, not described in *Acinetobacter*; ^5^ Ambler class A extended-spectrum ß-lactamase (ESBL) from *P. aeruginosa*, described in *Acinetobacter;* European Committee on Antimicrobial Susceptibility Testing, EUCAST; susceptible—dose-dependent susceptible—resistant, S-I-R; meropenem, MP; imipenem, IP.

## Data Availability

The data presented in this study are available on request from the corresponding author.

## Supplementary Materials

The following supporting information can be downloaded at: https://www.mdpi.com/article/10.3390/pathogens11040482/s1, Figure S1: Results of various incubation conditions of a 10 µg ertapenem disk in a 0.5 McFarland standard suspension of acquired carbapenemase-positive isolates (ACB10R, ACB11R) and the positive and negative study control strains; Table S1: Variations of incubation conditions of a 10 µg ertapenem disk in a 0.5 McFarland standard suspension of acquired carbapenemase-positive isolates ACB10R, ACB11R and the study control strains; Table S2: Sensitivity of various incubation conditions of a 10 µg ertapenem disk in a 0.5 McFarland standard suspension of acquired carbapenemase-positive isolates and a positive control strain (*n* = 3).
