# Supplementary material for: A Variant Carbapenem Inactivation Method (CIM) for Acinetobacter baumannii Group with Shortened Time-to-Result: rCIM-A
Source: Pathogens. 2022 Apr 18;11(4):482. doi: 10.3390/pathogens11040482 (PMC9024794; doi:10.3390/pathogens11040482)
Supplement: Supplementary file 1 [file pathogens-11-00482-s001.zip › pathogens-1681151-supplementary/pathogens-1639586_Figure S1.pdf]

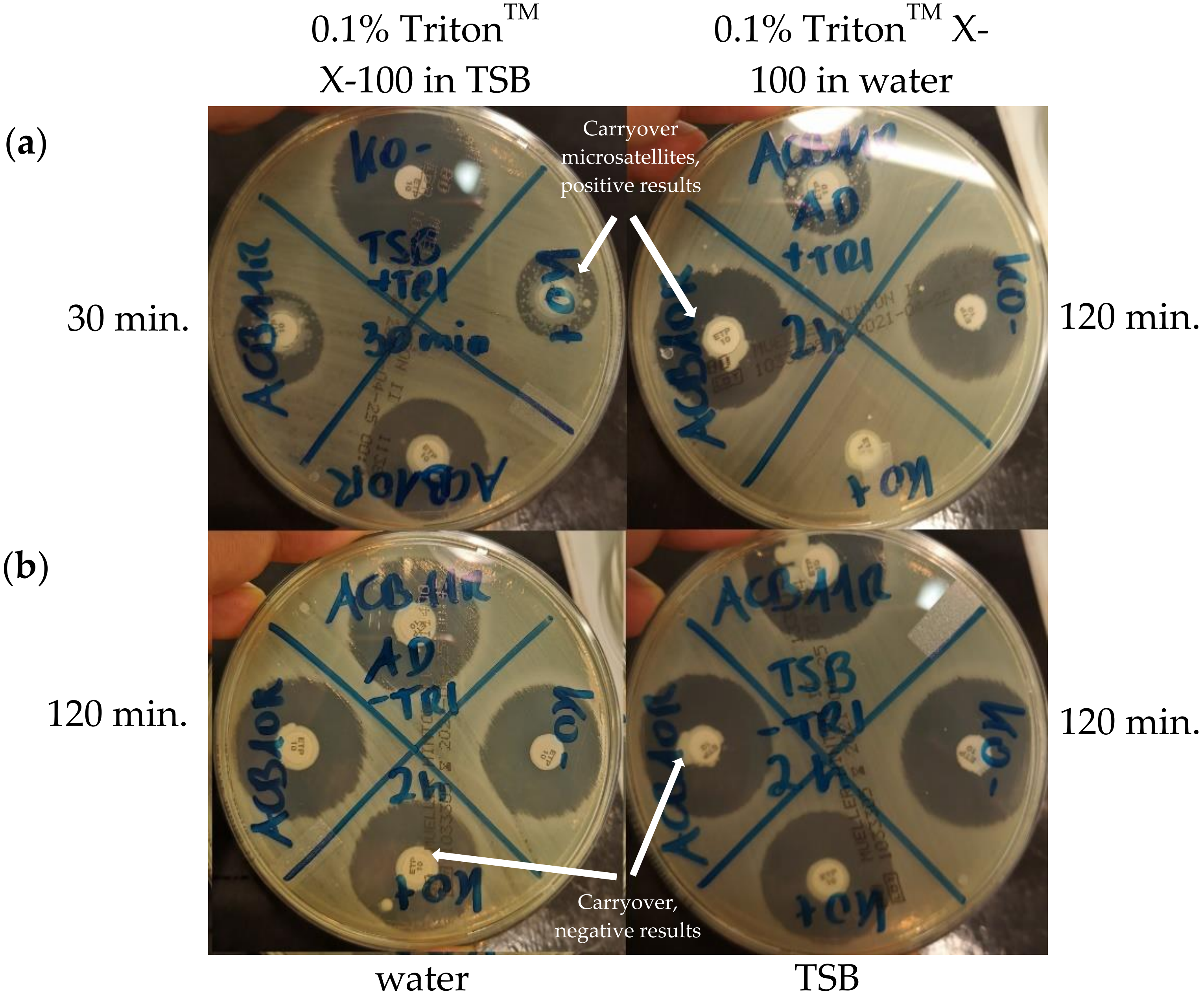

Figure S1. Results of various incubation conditions of a 10 μg ertapenem disk in a 0.5 McFarland standard suspension of acquired carbapenemase-positive isolates (ACB10R, ACB11R) and the positive and negative study control strains; **a.** suspensions without addition of 0.1% (vol/vol) Triton™ X-100 did not result in detection of carbapenemases; **b.** suspensions with addition of 0.1% (vol/vol) Triton™ X-100 enabled for detection of carbapenemase-positive strains; trypticase soy-broth, TSB; 0.1% (vol/vol) Triton™ X-100, TRI; water, AD; ertapenem 10 μg potency disk, ETP.
