# Supplementary material for: A Variant Carbapenem Inactivation Method (CIM) for Acinetobacter baumannii Group with Shortened Time-to-Result: rCIM-A
Source: Pathogens. 2022 Apr 18;11(4):482. doi: 10.3390/pathogens11040482 (PMC9024794; doi:10.3390/pathogens11040482)
Supplement: Supplementary file 1 [file pathogens-11-00482-s001.zip › pathogens-1681151-supplementary/pathogens-1639586_Table S1 and S2.pdf]

Table S1. Variations of incubation conditions of a 10 µg ertapenem disk in a 0.5 McFarland standard suspension of acquired carbapenemase-positive isolates ACB10R, ACB11R and the study control strains; tested conditions are marked by an"X".

Incubation-period (minutes)	Water	0.1% Triton™ X-100 in water	0.1% Triton™ X-100 in TSB	TSB
30		X		
60		X		
120	X	X	X	X

Trypticase soy-broth, TSB.

Table S2. Sensitivity of various incubation conditions of a 10 µg ertapenem disk in a 0.5 McFarland standard suspension of acquired carbapenemase-positive isolates and a positive control strain (n=3).

Incubation-period (minutes)	Water	0.1% Triton™ X-100 in water	0.1% Triton™ X-100 in TSB	TSB
30		1/3[1]		
60		1/3[1]		
120	0	3/3	3/3	0

[1] ACB10R not detected; trypticase soy-broth, TSB.
